# Relationship between Spatiotemporal Dynamics of the Brain at Rest and Self-Reported Spontaneous Thoughts: An EEG Microstate Approach

**DOI:** 10.3390/jpm11111216

**Published:** 2021-11-17

**Authors:** Povilas Tarailis, Dovilė Šimkutė, Thomas Koenig, Inga Griškova-Bulanova

**Affiliations:** 1 Life Sciences Center, Institute of Biosciences, Vilnius University, Sauletekio ave 7, LT-10257 Vilnius, Lithuania; povilas.tarailis@gmc.vu.lt (P.T.); dovile.simkute@gmc.vu.lt (D.Š.); 2 Translational Research Center, University Hospital of Psychiatry, University of Bern, Bolligenstrasse 111, CH-3000 Bern, Switzerland; thomas.koenig@upd.unibe.ch

**Keywords:** EEG, microstates, subjective experience

## Abstract

Rationale: The resting-state paradigm is frequently applied in electroencephalography (EEG) research; however, it is associated with the inability to control participants’ thoughts. To quantify subjects’ subjective experiences at rest, the Amsterdam Resting-State Questionnaire (ARSQ) was introduced covering ten dimensions of mind wandering. We aimed to estimate associations between subjective experiences and resting-state microstates of EEG. Methods: 5 min resting-state EEG data of 197 subjects was used to evaluate temporal properties of seven microstate classes. Bayesian correlation approach was implemented to assess associations between ARSQ domains assessed after resting and parameters of microstates. Results: Several associations between Comfort, Self and Somatic Awareness domains and temporal properties of neuroelectric microstates were revealed. The positive correlation between Comfort and duration of microstates E showed the strongest evidence (BF_10_ > 10); remaining correlations showed substantial evidence (10 > BF_10_ > 3). Conclusion: Our study indicates the relevance of assessments of spontaneous thought occurring during the resting-state for the understanding of the intrinsic brain activity reflected in microstates.

## 1. Introduction

Assessment of brain activity during the resting-state in clinical and cognitive neuroscience allows to shed some light on brain’s self-organization properties and how these patterns affect functioning and experiencing [1]. Functional magnetic resonance imaging (fMRI) and magneto- or electroencephalogram (MEG/EEG) studies revealed temporal dependences of neuronal activity patterns between anatomically separated brain regions at rest [2,3,4] that are affected in various brain disorders [5,6].

However, despite easy and straightforward implementation, the resting-state approach faces certain challenges. Two review papers [1,7] described the most common factors related to participants, procedures and measurements that should be taken into consideration during the resting-state studies. The control over the inner states of the participant is the most difficult one to address; although, it is worth mentioning that there are a few “resting-state” studies that implemented task-initiated thoughts [8,9,10,11]. Nevertheless, the knowledge on the relationships between subjective experiences and objectively observed physiological activity would help to improve the interpretation of the findings and increase the sensitivity and specificity of neuroimaging biomarkers in clinical and pharmacological studies [12]. In order to quantify subjects’ inner state, Diaz et al. introduced the Amsterdam Resting-State Questionnaire (ARSQ) as a tool to effectively quantify subjective experiences, emotions and thoughts during resting-state periods. The ARSQ covers ten resting-state cognition domains: Discontinuity of Mind (DoM), Theory of Mind (ToM), Self, Planning, Sleepiness, Comfort, Somatic Awareness (SA), Health Concerns (HC), Visual Thought (Vis) and Verbal Thought (VT) [12,13]. This tool has been successfully applied in neuroimaging studies, including those using EEG [14,15,16,17,18,19].

EEG signals can be analyzed using a variety of procedures, with microstate approach constituting one of the frequent ways to evaluate signal during the resting-state. The recorded oscillations are defined as the non-overlapping “states”, which are characterized by a unique spatial distribution [20] that reflect the functional cortical networks involved in the process and their temporal aspects. This way microstate approach offers a promising method to evaluate resting-state dynamics of the brain that can be further related to inner experiences. In a previous study, assessing four canonical microstates (A, B, C, D), Pipinis et al. [16] showed association between coverage of microstate C and ARSQ domain of Somatic Awareness. However, since then, the discussion regarding the estimation of the optimal number of microstates to extract has evolved [21,22,23]. It was noticed that topography of microstate classes differs between studies to a certain degree [23], with the most pronounced differences reported for microstate C: it displays anterior-posterior configuration in some studies and posterior maximum—in others. Custo et al. showed that when only four microstate classes are extracted, spatially similar but functionally different microstates might be merged into a single microstate [22]; this potentially could explain the functional heterogeneity of microstate C reported in the literature. Based on the visual inspection, microstate C from the study by Pipinis and colleagues [16] had more occipital-parietal pattern of activation than the canonical microstate C (anterior-posterior), and was more topographically similar to microstate F reported in the later studies extracting more than four canonical microstates [21,22].

In this study, we extracted the optimal number of microstates for the dataset and estimated spatial correlations between microstate topographies in an attempt to distinguish spatially similar microstates (C and F). We aimed to test whether Somatic Awareness is related to microstate F rather than microstate C. We also evaluated the association between biologically defined microstate activity of other classes and different subjective experiences and emotions occurring during the resting-state session as measured with ARSQ. We relied on the Bayesian approach for the interpretation of the observed correlations due to its better accuracy in small samples, less proneness to type I errors, and straightforward interpretation of result [24].

## 2. Materials and Methods

### 2.1. Participants

202 participants, aged from 19 to 35 years, with normal or corrected-to-normal vision were included. All subjects were Caucasians residing in Lithuania. Eighteen participants were left-handed and the remaining 184 were right-handed. 107 healthy, non-pregnant, not using hormonal contraception and experiencing regular menstrual cycle females were recruited. Based on self-reports, 82 females participated in the study during the early follicular phase (menses), 21—during the luteal phase and 4—during the ovulatory phase. Part of the data (*n* = 94) was used in the previous report [16]. All subjects gave their written informed consent to participate, and the study was approved by the Vilnius Regional Biomedical Research Ethics Committee (Nr.2019/10-1159-649, date of approval: 8 October 2019). Subjects with any reported neurological and/or psychiatric disorders, any kind of addiction or the use of psychotropic substances were excluded. Participants were asked not to use nicotine and caffeine 2 h prior to the study.

### 2.2. Data Collection

For the EEG collection, subjects were comfortably seated in the upright position in a dim lighted, sound attenuated and electrically shielded room. Before the start of the session participants were instructed to keep their eyes closed, not to think about anything in particular and not to fall asleep. Five minutes of eyes-closed resting-state EEG data were collected using a 64 channels WaveGuard EEG (International 10-10 System) cap with silver/silver chloride electrodes and EEG equipment (ANT Neuro, Hangelo, The Netherlands). Two additional electrodes pairs (VEOG and HEOG) were placed above and below the right eye and the right and left outer canthi to record vertical and horizontal eye movements. All electrodes were referenced against mastoids. Impedance was kept below 20 kΩ. The sampling rate of 512 Hz [16] and 2048 Hz were used. Right after the EEG recording session participants completed the Lithuanian version of ARSQ, where they had to rate the statements about the emotions and thoughts during the session on Likert-type scale ranging from 1 (completely disagree) to 5 (completely agree).

In total, data from five participants were excluded from further analysis: one due to family psychiatric history and four due to incomplete ARSQ, resulting in a total of 197 participants (F = 103, M = 94, age 23.97 ± 3.81).

### 2.3. ARSQ

The Lithuanian version of ARSQ 2.0 was used [16]. The ARSQ contains 54 statements on thoughts and feelings that participants may experience during resting-state period. Each statement is rated on Likert-type scale ranging from 1 (completely disagree) to 5 (completely agree). The ARSQ covers ten different domains of resting-state cognition: Discontinuity of Mind, Theory of Mind, Self, Planning, Sleepiness, Comfort, Somatic Awareness, Health Concern, Visual Thought and Verbal Thought. Each domain is evaluated with three statements. The scores of each ARSQ dimension were calculated by taking the mean value of three statements.

### 2.4. EEG Processing

The offline EEG data processing was conducted in MATLAB (The Mathworks, Natick, USA) environment using EEGLAB toolbox [25]. 50 Hz power line noise was removed using the Thomas F-statistics implemented in the CleanLine plugin for EEGLAB [26]. The artefacts caused by vertical and horizontal eye movements and cardiac pulse were corrected using an ICA approach [27]. Channels with excessive artefacts were manually rejected and reconstructed using spherical spline method [28]. Data were segmented into artefact-free, non-overlapping 2 s epochs, resulting in average of 144.48 epochs per record. Data were downsampled to 512 Hz, recomputed to average reference and filtered between 1 and 40 Hz using Butterworth filter of 2nd order.

### 2.5. Microstate Analysis

The microstate analysis was performed using microstate plugin for EEGLAB (version v1.2) (http://www.thomaskoenig.ch/index.php/software/microstates-in-eeglab/). Clusterization for the microstate analysis was performed in two steps: first, the maps at momentary peaks of the global field power were extracted and submitted to modified k-means clustering algorithm [29]. To ensure that only spatial distribution of these maps was taken into account, the maps were normalized to a vector of length 1 and polarity ignored. To identify the optimal number of microstate templates, number of *k* ranged from 2 to 10. To maximize global explained variance clusterization was repeated 50 times for each number of *k*. For the second step, individual topographies were averaged across participants using a permutation algorithm that maximizes the common variance between the participants [30]. The optimal number of *k* for grand average was identified by Silhouettes method [31], which evaluates how similar each data point (individual topography) is to other data points in its own cluster compared to the data points in other clusters [21,32]. Silhouette values are defined as:

S = (b_i_ − a_i_)/max (a_i_, b_i_) (1)

where a_i_ is the average distance from i-th point to other points in the same cluster, and b_i_ is the average distance between i-th point and points in different clusters. As for the measure of distance, Global Map Dissimilarity (GMD) was applied as it is described in literature [33]. The distances between data points are inversely proportional to the similarity among the corresponding datapoints (i.e., low GMD—high similarity).

Group level topographies were backfitted to individual EEGs by winner-takes-all approach [23,34]. Duration (Dur), occurrence (Occ), coverage (Cov) and global field power (GFP) measures were calculated for each microstate class. To quantify the spatial similarity between microstate topographies, we calculated spatial correlation. To ensure that polarity of topographies was ignored, the absolute values of spatial correlation were taken.

### 2.6. Statistical Analysis

A Bayesian Pearson correlation coefficients, and the corresponding Bayes factors (BF) were computed between coverage estimates of microstate F and microstate C and ARSQ domain of Somatic Awareness. The Bayesian Pearson correlation was also used to explore the associations of each ARSQ domain with the EEG features (duration, occurrence, coverage and GFP) other than specifically defined in the hypothesis.

In order to expand the knowledge in the field, we assessed potential effects of age and gender on both ARSQ scores and microstate parameters. The multivariate ANOVA with gender as a fixed factor and age as covariate was used for ARSQ scores. The two-way repeated measure ANOVA (microstate × gender) were run separately for each microstate parameter (duration, occurrence, coverage and GFP) with age as covariate. Significant age effects were followed with Bayesian Pearson correlation, and significant gender effects were followed by a Bonferroni post-hoc test. Additionally, intraclass Bayesian Pearson correlation coefficients were calculated between ARSQ dimensions.

The correlations were computed using JASP statistical software (Version 0.14.1) [35,36]. In Bayesian interference, probability is a measure of the degree of confidence in the occurrence of an event and instead of p value it provides a likelihood ratio—Bayes factor (BF), the predictive updating factor which measures the change in relative beliefs about the alternative hypothesis relative to the null hypothesis [37] and does not require correction for multiple comparisons [38,39,40]. BF can state evidence for both the alternative and the null hypothesis. Evidence categories for the BF are divided into eleven categories: Decisive evidence for H1 (BF_10_ > 100); Very strong evidence for H1 (100 > BF_10_ > 30); Strong evidence for H1 (30 > BF_10_ > 10); Substantial evidence for H1 (10 > BF_10_ > 3); Anecdotal evidence for H1 (3 > BF_10_ > 1); No evidence (BF_10_ = 1); Anecdotal evidence for H0 (1 > BF_10_ > 1/3); Substantial evidence for H0 (1/3 > BF_10_ > 1/10); Strong evidence for H0 (1/10 > BF_10_ > 1/30); Very strong evidence for H0 (1/30 > BF_10_ > 1/100); Decisive evidence for H0 (BF_10_ < 1/100) [41,42]. *p* value of 0.05 corresponds to BF in favor of the alternative hypothesis at 2.44 [43].

## 3. Results

### 3.1. EEG Microstates

**Figure 1:**
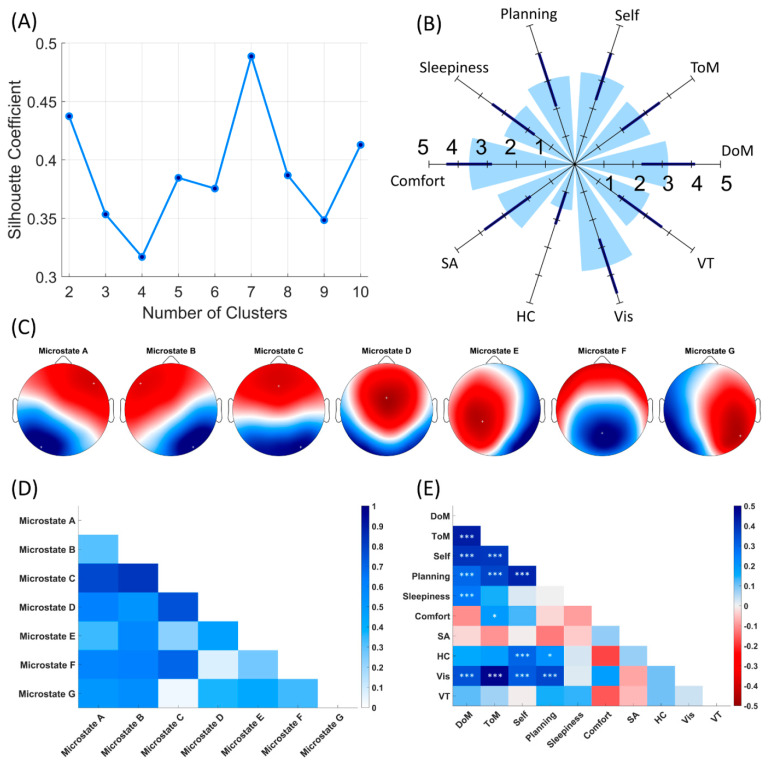
(**A**) Optimal number of microstates based on the Silhouette Coefficient values for each number of *k*; number of estimated clusters equals 7. (**B**) Mean scores of each ARSQ dimension. Dark blue lines indicate standard deviation. (**C**) Seven microstate group-level topographies. (**D**) Spatial correlation coefficients between group-level topographies. (**E**) Intraclass Bayesian Pearson correlation coefficients between ARSQ dimensions. * 10 > BF_10_ > 3, *** BF_10_ > 100.

Silhouette coefficient (mean value of silhouettes for each number of *k*) yielded a maximum value at *k* = 7 (Figure 1A). Four microstate topographies with right frontal to left posterior, left frontal to right posterior, frontal to occipital and fronto-central configurations matched the most frequently reported microstate classes in the literature and were labeled as microstates A, B, C and D, respectively [44]. Among three additional topographies, one had left lateralized activity and was similar to microstate E reported in studies by [21,22,45] and was labeled accordingly as microstate E. One topography displayed posterior activity and matched microstate F reported in studies by [21,22,46], microstate E reported in studies by [47,48,49] and microstate C′ reported in study [50] and was further labeled as microstate F. The remaining topography had right lateralized activity and was similar to microstate G from study by [22] and microstate F reported in studies by [45,49]; this was further labeled as microstate G (Figure 1C). The extracted seven microstates explained 83.8% of the global variance. Means and standard deviations of the temporal characteristics of each of seven microstates are presented in Table 1. The parameters of extracted microstates are in the range of those reported in the literature [23,51,52].

To further compare microstates to previously published results, the potential age and gender effects were tested using two-way ANOVAs with gender set as a fixed factor and age as covariate separately for each microstate measure. For the duration, a significant effect of age [F(1, 194) = 3.926, *p* = 0.049] and gender [F(1, 194) = 4.380, *p* = 0.038] was observed. Follow-up analysis revealed that only the correlation between age and the duration of microstate D reached the substantial level of evidence (*r* = 0.201, BF_10_ = 4.852), and that males had longer microstate durations than females. For the occurrence, a significant effect of age was revealed [F(1, 194) = 4.432, *p* = 0.037]; however, only a negative correlation between the occurrence of microstate A and age that reached the strong level of evidence (*r* = −0.224, BF_10_ = 12.761). No effect of either age or gender was observed on the coverage measures. For GFP, gender effect [F(1, 194) = 9.620, *p* = 0.002], and a significant interaction between gender and microstate class [F(6, 194) = 3.291, *p* = 0.018] was observed; overall males had lower GFPs than females. However, comparison between genders was nonsignificant on the Bonferroni post hoc test for all the microstates.

The full tables with all ANOVAs results and Bayesian Pearson correlation outcomes are presented in the Supplementary Tables S1–S6.

### 3.2. Subjective Reports

Mean scores and standard deviations for the scores on each ARSQ dimensions were as follows: DoM 3.273 (0.936), ToM 2.846 (0.823), Self 3.228 (0.867), Planning 3.010 (0.973), Sleepiness 2.668 (0.924), Comfort 3.706, (0.801), SA 2.914 (1.002), HC 1.616 (0.591), Vis 3.760 (1.015), VT 2.821 (0.952). These are summarized in polar chart in Figure 1B. Intraclass Bayesian Pearson correlation coefficients for ARSQ dimensions are displayed in Figure 1E. To account for potential age and gender effects, the effect of the fixed factor gender on the ARSQ scores with age as covariate were tested using multivariate ANOVA. Multivariate ANOVA revealed a significant main effect of the covariate age for ARSQ scores [F(10, 185) = 2.502, *p* = 0.008], but no effect of gender was observed [F(10, 185) = 1.348, *p* = 0.208]. A subsequent correlation analysis showed that only correlations between age and ToM and HC domains reached substantial level of evidence (ToM: *r* = −0.193, BF_10_ = 3.520, Health: *r* = −0.198, BF_10_ = 4.258).

### 3.3. Association between Temporal Parameters of Microstates and ARSQ Dimensions

Bayesian Pearson correlation showed a negative association between coverage of microstate F and Somatic awareness (*r* = −0.210, BF_10_ = 6.871) and no correlation between coverage of microstate C and Somatic awareness (*r* = −0.007, BF_10_ = 0.090), confirming our initial hypothesis.

Additionally, Bayesian Pearson correlation showed significant interaction between Self domain and duration of microstate D (*r* = −0.203, BF_10_ = 5.224) and occurrence of microstate B (*r* = 0.192, BF_10_ = 3.305). Bayesian Pearson correlation also revealed a negative relationship with the occurrence of microstate C (*r* = −0.212, BF_10_ = 7.638) and positive relationships with duration of microstate E (*r* = 0.220, BF_10_ = 10.949) and duration of microstate G (*r* = 0.203, BF_10_ = 5.284).

**Figure 2:**
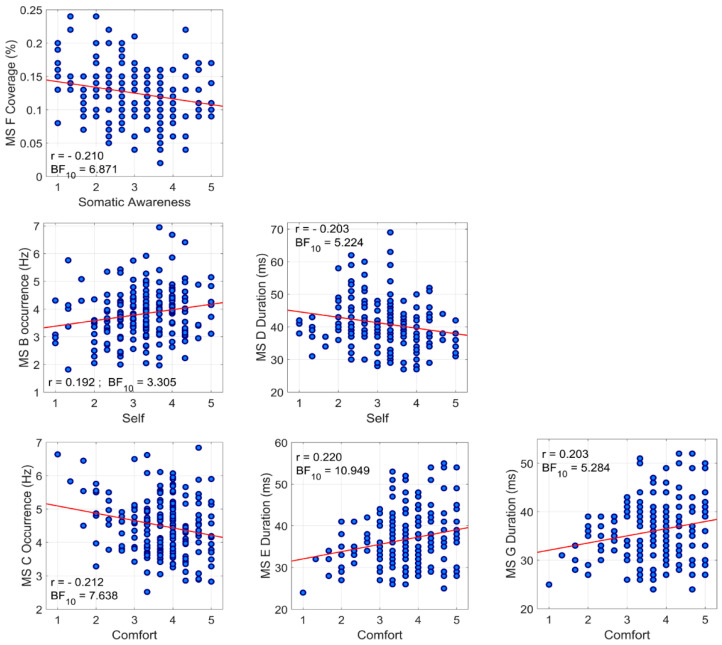
Scatter plots displaying relationship between microstate parameters and Amsterdam Resting-State Questionnaire (ARSQ) dimensions.

Bayesian Pearson correlation coefficients for temporal characteristics of each microstate class and scores of ARSQ dimensions are summarized in Table 2. Significant associations between microstate characteristics and ARSQ domains are presented in scatter plots in Figure 2.

## 4. Discussion

The relationship between subjective experiences at rest and brain functional activity is not well known. In this study we aimed to evaluate the association of subjective experiences as measured with Amsterdam Resting-State Questionnaire and temporal properties of the resting-state EEG assessed with the microstate approach. The microstate approach allows evaluation of rapidly changing network reorganization that occurs in order to mediate complex mental activities and optimally respond to rapidly changing input. We paid particular attention to microstate classes C and F that can be frequently merged into one when a suboptimal number of microstate is extracted, and expected that parameters of class F would be related to Somatic Awareness domain in a manner similar to microstate C as observed previously by [16].

An initial work by Pipinis et al. [16] related ARSQ to the parameters of four classical microstates (A, B, C, D), showing a negative association between Somatic Awareness (which is evaluated with statements ‘I was conscious of my body’, ‘I thought about my heartbeat’, ‘I thought about my breathing’) and coverage of microstate C. Lately, Zanesco et al. [18], performed a meta analytic correlation combining results by Pipinis et al. and their own results and showed the inverse association between ARSQ domain of Somatic Awareness and a coverage of microstate C. However, Custo et al. demonstrated that when only four microstates are extracted, the functionally different but spatially overlapping microstates—C and F—are merged into a single microstate C [22]. Thus, in the current work, we estimated the optimal data-driven number of microstates without putting a priori constraint on the number to be extracted. The optimal number of microstate configurations was estimated at 7, corresponding to the report by [22]. Importantly, the spatial correlation between microstates C and F was ~0.7 (Figure 1D), thus supporting the results of Custo et al. [22] that the functionally different but spatially overlapping microstates—C and F—might be merged into a single microstate C [22]. This is further supported by the spatial correlation observed between microstate C from *k* = 4 with microstates C and F from *k* = 7 (result presented in the Supplementary Figure S1).

We suggest that a negative correlation observed between the coverage of microstate F and the dimension of Somatic Awareness in the current work resembles the same associations as observed by Pipinis et al. and Zanesco et al. [16,18]. Custo et al. reported the strongest activity for microstate F in the dorsal anterior cingulate cortex, superior frontal gyrus, middle frontal gyrus and insula. These loci overlap with the Salience network and correspond to the sources of the microstate C reported by Britz and colleagues [53]. Britz et al. [53] associated microstate C with integration of interoceptive information with emotional salience. Several studies extracting four canonical microstates reported increased microstate C activity during the resting-state period compared to different tasks [11,54,55] and associated it with task-negative Default Mode Network (DMN) [21,22]. Likely, part of this involvement is also seen as the negative association between the occurrence of microstate C and the Comfort (measured with questions like ‘I felt comfortable’, ‘I felt relaxed’, ‘I felt happy’) in our study. The ability to relax and feel comfortable is related to interoceptive aspects through the urge to restore balance in physical and emotional context [56,57]. The domain of Comfort was previously related with the ability to switch between tasks [58], correlated with character traits of self-directedness (associated with individual ability to govern behavior according to situational demand) [13] and mental and physical well-being [12].

To note, the Comfort domain of ARSQ was also positively associated with the duration of microstates E and G. However, due to the fact that we had no a priori expectations regarding microstate classes other than C and F, these correlations should be regarded as exploratory. Nevertheless, we attempt to discuss the observed correlations in the framework of known functional aspects behind certain microstate classes.

Microstate E is a relatively newly described microstate and it was reported only in recent studies. Brechet and colleagues [21] found the main sources of microstate E in the right medial prefrontal cortex that is a subsystem of DMN, which participates in the theory of mind and mental simulations [59] and Custo et al. [22] observed the strongest activity for microstate E in the anterior cingulate cortex, posterior cingulate cortex and precuneus—these areas are parts of DMN. Thus, an opposite relationship between domain of Comfort and microstates C and E probably reflects the different aspects of DMN involvement that is known to play a crucial role in processing of personally significant information, self-reflection and self-referential internal mentation [60].

Microstate G was reported only in four studies so far [22,45,49,61] and the functional role of this microstate remains unclear. Custo et al. [22] localized sources for microstate G in the right inferior parietal lobe, superior temporal gyrus and cerebellum and associated microstate G with the sensorimotor network due to the strong activity observed in cerebellum. Stoffers and colleagues [19] reported a positive correlation between ARSQ domain of Comfort and functional connectivity within Sensorimotor network. Thus, the observed positive association between duration of microstate G and Comfort potentially reflects the physical aspect of well-being stemming from appropriate somatosensory activation.

Finally, the ARSQ domain of Self was positively correlated to the occurrence of microstate B and negatively correlated to the duration of microstate D. Although microstate B was previously associated with verbalization [10], visual processing [11,46], and activity in the visual network [22,53], Bréchet et al., [21] related microstate B with conscious experience, autobiographic memory, visualization of the scene and visualization of the self in the scene. Vellante et al. [62] reported a negative association between microstate B and states of dissociation and anxiety in bipolar patients, interpreting results as reflecting autobiographic memory deficits and increased self-focusing. ARSQ dimension of Self is evaluated with statements ‘I thought about my feelings’, ‘I thought about my behavior’ and ‘I thought about myself’. The last two statements are particularly intriguing in the context of findings by Brechet et al. [21] and Vellante et al. [62], since they both are directly related to autobiographic memory and self-visualization in the particular scene. It is possible that the observed relationship between microstate B and domain of Self reflects the aspects of self-visualization.

While domain of Self is orientated towards the inner mentation, microstate D is associated to externally orientated processing [63]. Several studies reported an increased activity of microstate D during various tasks and states and associated it with attention attributes, working memory, cognitive control, detecting behaviorally relevant stimuli [11,21,46,55] and fronto-parietal network [22,53]. Thus, our observation on the opposite relationship between Self domain and microstates B and D probably reflects the dissociation from external environment during the resting-state with closed eyes.

It should be noted that the observed correlations were not strong, but in the range of the reported strengths for associations between physiological and psychological variables. It has also been suggested that correlations are highly variable between studies in small sample sizes (*n* < 250) [64]. Our sample is the largest up to date, where microstate assessment was performed alongside ARSQ. However, it is important that future studies on individual differences include larger sample sizes, so they are powered to detect small to moderate correlations between ARSQ ratings and microstates.

To further advance the current knowledge, we attempted to evaluate gender and age influence on both ARSQ domains and microstate parameters. The significant main effect of the covariate age for ARSQ scores was observed, but only negative associations between age and ToM and HC domains reached substantial level of evidence. Previously, Diaz et al. [13] reported negative correlations between age and DoM, Self, Planning, Visual and Verbal Thoughts. However, their sample was more diverse in respect to age (19–85 years) and this might have contributed to the discrepancy in the results. In line with Diaz et al. we did not find any gender effect on ARSQ. A limited number of studies up to date have addressed the question of gender- and age-related differences in microstate parameters. Tomescu et al. [65] reported higher occurrence rate of microstate D for males, while duration of microstate C was longer for females. Moreover, they did not find any differences in occurrence rate for microstates A, B and C, but reported higher rate of microstate class D for males. Zanesco et al. [52] reported shorter mean durations of microstates A, B, D, and E and more frequent occurrences of microstates A, B, and C in males. In our study, males had longer durations of microstates and lower GFPs; however, both Tomescu et al. [65] and Zanesco et al. [52] worked with groups of wide age distribution in contrast to our sample that was homogenous in respect to age. This could also explain why we observed only few associations between age and microstate parameters. In our cohort, only the positive correlation between age and the duration of microstate D and a negative correlation between age and the occurrence of microstate A reached the substantial-to-strong level of evidence. These results are partially in line with those reported by Koenig et al. [44]. The authors reported shorter duration of microstate D during early (12–16 year) and late adolescence (16–21 years) and a partial reverse of this tendency in adulthood (above 21 years). Also, microstate A was shorter and had lower occurrence rate in adulthood compared to late adolescence.

## 5. Conclusions

In this study, we showed that microstate C and F are strongly spatially correlated and potentially merging when four classical microstates are extracted. Importantly, these two microstates display a distinct correlation pattern with different subjective experiences during the resting-state period. Microstate F rather than microstate C was associated to the Somatic Awareness. Microstates C, E, and G were related to the scores on the Comfort domain, while microstates B and D were related to the Self domain. Our study indicates the relevance of assessments of spontaneous thought occurring during the resting-state for understanding of the intrinsic brain activity reflected in microstates.

## Figures and Tables

**Table 1:** Temporal parameters of microstates.

	Duration (ms)	Occurrence (Hz)	Coverage (%)
MS A	43.361 (±8.26)	3.6 (±0.95)	15.13 (±4)
MS B	45.337 (±9.25)	3.8 (±0.89)	16.93 (±4.2)
MS C	52.505 (±14.18)	4.5 (±0.83)	22.91 (±6.5)
MS D	40.909 (±7.16)	3.44 (±1)	13.82 (±3.9)
MS E	36.718 (±6.31)	2.62 (±0.73)	9.43 (±2.4)
MS F	39.589 (±7.34)	3.22 (±0.99)	12.56 (±4)
MS G	36.056 (±5.81)	2.65 (±0.81)	9.31 (±2.5)

**Table 2:** Bayesian Pearson correlation coefficients between microstate characteristics and ARSQ scores.

		DoM	ToM	Self	Planning	Sleep	Comfort	SA	Health	Visual	Verbal
**Microstate A**	**Dur**	*r* = −0.020 BF_10_ = 0.093	*r* = 0.029 BF_10_ = 0.097	*r* = −0.119 BF_10_ = 0.352	*r* = −0.008 BF_10_ = 0.090	*r* = −0.005 BF_10_ = 0.089	*r* = 0.134 BF_10_ = 0.512	*r* = 0.034 BF_10_ = 0.100	*r* = −0.018 BF_10_ = 0.092	*r* = 0.023 BF_10_ = 0.099	*r* = −0.037 BF_10_ = 0.102
	**Occ**	*r* = 0.012 BF_10_ = 0.090	*r* = 0.051 BF_10_ = 0.115	*r* = 0.096 BF_10_ = 0.220	*r* = −0.038 BF_10_ = 0.103	*r* = 0.111 BF_10_ = 0.294	*r* = −0.082 BF_10_ = 0.172	*r* = −0.019 BF_10_ = 0.092	*r* = 0.028 BF_10_ = 0.096	*r* = 0.062 BF_10_ = 0.129	*r* = 0.035 BF_10_ = 0.100
	**Cov**	*r* = −0.009 BF_10_ = 0.090	*r* = 0.056 BF_10_ = 0.121	*r* = 0.010 BF_10_ = 0.090	*r* = −0.055 BF_10_ = 0.119	*r* = 0.097 BF_10_ = 0.223	*r* = 0.002 BF_10_ = 0.089	*r* = −0.005 BF_10_ = 0.089	*r* = 0.050 BF_10_ = 0.114	*r* = 0.056 BF_10_ = 0.120	*r* = 0.030 BF_10_ = 0.097
	**GFP**	*r* = −0.016 BF_10_ = 0.091	*r* = 0.040 BF_10_ = 0.104	*r* = −0.066 BF_10_ = 0.137	*r* = −0.039 BF_10_ = 0.103	*r* = −0.015 BF_10_ = 0.091	*r* = 0.115 BF_10_ = 0.324	*r* = 0.001 BF_10_ = 0.089	*r* = −0.027 BF_10_ = 0.096	*r* = 0.037 BF_10_ = 0.102	*r* = −0.060 BF_10_ = 0.127
**Microstate B**	**Dur**	*r* = −0.028 BF_10_ = 0.096	*r* = 0.028 BF_10_ = 0.096	*r* = −0.054 BF_10_ = 0.119	*r* = 0.052 BF_10_ = 0.116	*r* = −0.101 BF_10_ = 0.242	*r* = 0.182 BF_10_ = 2.339	*r* = −0.004 BF_10_ = 0.089	*r* = −0.063 BF_10_ = 0.131	*r* = 0.012 BF_10_ = 0.090	*r* = −0.016 BF_10_ = 0.091
	**Occ**	*r* = 0.068 BF_10_ = 0.139	*r* = −0.017 BF_10_ = 0.092	*r* = 0.192 BF_10_ = 3.305	*r* = 0.083 BF_10_ = 0.173	*r* = −0.038 BF_10_ = 0.103	*r* = −0.117 BF_10_ = 0.337	*r* = 0.024 BF_10_ = 0.094	*r* = 0.044 BF_10_ = 0.107	*r* = −0.003 BF_10_ = 0.089	*r* = 0.062 BF_10_ = 0.130
	**Cov**	*r* = 0.038 BF_10_ = 0.103	*r* = 0.008 BF_10_ = 0.090	*r* = 0.130 BF_10_ = 0.464	*r* = 0.111 BF_10_ = 0.295	*r* = −0.121 BF_10_ = 0.372	*r* = 0.048 BF_10_ = 0.111	*r* = −0.001 BF_10_ = 0.089	*r* = 0.004 BF_10_ = 0.089	*r* = 0.006 BF_10_ = 0.090	*r* = 0.058 BF_10_ = 0.123
	**GFP**	*r* = −0.019 BF_10_ = 0.092	*r* = 0.040 BF_10_ = 0.104	*r* = −0.058 BF_10_ = 0.124	*r* = −0.019 BF_10_ = 0.092	*r* = −0.041 BF_10_ = 0.105	*r* = 0.128 BF_10_ = 0.442	*r* = 0.008 BF_10_ = 0.090	*r* = −0.034 BF_10_ = 0.099	*r* = 0.039 BF_10_ = 0.104	*r* = −0.076 BF_10_ = 0.155
**Microstate C**	**Dur**	*r* = −0.070 BF_10_ = 0.141	*r* = −0.028 BF_10_ = 0.096	*r* = −0.124 BF_10_ = 0.398	*r* = −0.063 BF_10_ = 0.131	*r* = −0.003 BF_10_ = 0.089	*r* = 0.106 BF_10_ = 0.266	*r* = 0.008 BF_10_ = 0.090	*r* = −0.050 BF_10_ = 0.113	*r* = −0.045 BF_10_ = 0.109	*r* = −0.067 BF_10_ = 0.138
	**Occ**	*r* = 0.004 BF_10_ = 0.089	*r* = −0.028 BF_10_ = 0.096	*r* = 0.093 BF_10_ = 0.205	*r* = −0.042 BF_10_ = 0.106	*r* = 0.074 BF_10_ = 0.151	*r* = −0.212 BF_10_ = 7.638	*r* = −0.021 BF_10_ = 0.093	*r* = 0.034 BF_10_ = 0.100	*r* = −0.023 BF_10_ = 0.094	*r* = −0.006 BF_10_ = 0.089
	**Cov**	*r* = −0.056 BF_10_ = 0.120	*r* = −0.046 BF_10_ = 0.109	*r* = −0.059 BF_10_ = 0.125	*r* = −0.100 BF_10_ = 0.237	*r* = 0.027 BF_10_ = 0.096	*r* = −0.037 BF_10_ = 0.102	*r* = −0.007 BF_10_ = 0.090	*r* = −0.011 BF_10_ = 0.090	*r* = −0.047 BF_10_ = 0.110	*r* = −0.063 BF_10_ = 0.130
	**GFP**	*r* = −0.023 BF_10_ = 0.094	*r* = 0.035 BF_10_ = 0.101	*r* = −0.070 BF_10_ = 0.144	*r* = −0.038 BF_10_ = 0.103	*r* = −0.030 BF_10_ = 0.097	*r* = 0.112 BF_10_ = 0.302	*r* = 0.009 BF_10_ = 0.090	*r* = −0.032 BF_10_ = 0.099	*r* = −0.033 BF_10_ = 0.99	*r* = −0.081 BF_10_ = 0.169
**Microstate D**	**Dur**	*r* = 0.069 BF_10_ = 0.141	*r* = −0.034 BF_10_ = 0.100	*r* = −0.203 BF_10_ = 5.224	*r* = −0.037 BF_10_ = 0.102	*r* = −0.101 BF_10_ = 0.239	*r* = 0.177 BF_10_ = 1.939	*r* = 0.103 BF_10_ = 0.247	*r* = −0.067 BF_10_ = 0.138	*r* = −0.041 BF_10_ = 0.105	*r* = −0.048 BF_10_ = 0.111
	**Occ**	*r* = −0.009 BF_10_ = 0.090	*r* = −0.054 BF_10_ = 0.119	*r* = 0.008 BF_10_ = 0.090	*r* = −0.088 BF_10_ = 0.189	*r* = 0.094 BF_10_ = 0.210	*r* = −0.128 BF_10_ = 0.438	*r* = 0.135 BF_10_ = 0.531	*r* = 0.054 BF_10_ = 0.118	*r* = 0.024 BF_10_ = 0.094	*r* = 0.037 BF_10_ = 0.102
	**Cov**	*r* = −0.057 BF_10_ = 0.122	*r* = −0.078 BF_10_ = 0.160	*r* = −0.115 BF_10_ = 0.322	*r* = −0.100 BF_10_ = 0.234	*r* = 0.024 BF_10_ = 0.094	*r* = −0.031 BF_10_ = 0.098	*r* = 0.158 BF_10_ = 1.034	*r* = 0.026 BF_10_ = 0.095	*r* = −0.053 BF_10_ = 0.117	*r* = 0.020 BF_10_ = 0.093
	**GFP**	*r* = −0.015 BF_10_ = 0.091	*r* = 0.026 BF_10_ = 0.095	*r* = −0.093 BF_10_ = 0.206	*r* = −0.043 BF_10_ = 0.107	*r* = −0.024 BF_10_ = 0.094	*r* = 0.121 BF_10_ = 0.371	*r* = 0.039 BF_10_ = 0.103	*r* = −0.041 BF_10_ = 0.105	*r* = 0.033 BF_10_ = 0.099	*r* = −0.076 BF_10_ = 0.155
**Microstate E**	**Dur**	*r* = −0.040 BF_10_ = 0.104	*r* = 0.016 BF_10_ = 0.091	*r* = −0.116 BF_10_ = 0.328	*r* = 0.043 BF_10_ = 0.107	*r* = −0.108 BF_10_ = 0.279	*r* = 0.220 BF_10_ = 10.95	*r* = 0.056 BF_10_ = 0.121	*r* = −0.083 BF_10_ = 0.172	*r* = 0.011 BF_10_ = 0.090	*r* = −0.045 BF_10_ = 0.108
	**Occ**	*r* = 0.025 BF_10_ = 0.095	*r* = −0.015 BF_10_ = 0.091	*r* = 0.116 BF_10_ = 0.329	*r* = 0.015 BF_10_ = 0.091	*r* = −0.110 BF_10_ = 0.090	*r* = −0.081 BF_10_ = 0.167	*r* = 0.034 BF_10_ = 0.099	*r* = 0.030 BF_10_ = 0.097	*r* = 0.026 BF_10_ = 0.095	*r* = 0.008 BF_10_ = 0.090
	**Cov**	*r* = 0.015 BF_10_ = 0.091	*r* = −0.030 BF_10_ = 0.097	*r* = 0.030 BF_10_ = 0.097	*r* = 0.037 BF_10_ = 0.102	*r* = −0.110 BF_10_ = 0.289	*r* = 0.052 BF_10_ = 0.116	*r* = 0.067 BF_10_ = 0.137	*r* = −0.027 BF_10_ = 0.096	*r* = 0.020 BF_10_ = 0.093	*r* = 0.001 BF_10_ = 0.089
	**GFP**	*r* = −0.015 BF_10_ = 0.091	*r* = 0.037 BF_10_ = 0.102	*r* = −0.066 BF_10_ = 0.137	*r* = −0.016 BF_10_ = 0.092	*r* = −0.034 BF_10_ = 0.100	*r* = 0.135 BF_10_ = 0.530	*r* = 0.021 BF_10_ = 0.093	*r* = −0.040 BF_10_ = 0.104	*r* = 0.040 BF_10_ = 0.104	*r* = −0.077 BF_10_ = 0.158
**Microstate F**	**Dur**	*r* = −0.038 BF_10_ = 0.103	*r* = 0.014 BF_10_ = 0.091	*r* = −0.134 BF_10_ = 0.507	*r* = 0.088 BF_10_ = 0.188	*r* = −0.030 BF_10_ = 0.097	*r* = 0.121 BF_10_ = 0.374	*r* = −0.101 BF_10_ = 0.241	*r* = −0.093 BF_10_ = 0.205	*r* = −0.009 BF_10_ = 0.090	*r* = −0.022 BF_10_ = 0.093
	**Occ**	*r* = 0.051 BF_10_ = 0.115	*r* = 0.029 BF_10_ = 0.097	*r* = 0.071 BF_10_ = 0.145	*r* = 0.119 BF_10_ = 0.352	*r* = 0.082 BF_10_ = 0.172	*r* = −0.111 BF_10_ = 0.294	*r* = −0.140 BF_10_ = 0.607	*r* = −0.026 BF_10_ = 0.095	*r* = 0.026 BF_10_ = 0.092	*r* = −0.018 BF_10_ = 0.092
	**Cov**	*r* = 0.035 BF_10_ = 0.100	*r* = 0.041 BF_10_ = 0.105	*r* = −0.016 BF_10_ = 0.092	*r* = 0.146 BF_10_ = 0.710	*r* = 0.043 BF_10_ = 0.107	*r* = −0.036 BF_10_ = 0.101	*r* = −0.210 BF_10_ = 6.871	*r* = −0.064 BF_10_ = 0.132	*r* = 0.019 BF_10_ = 0.092	*r* = −0.031 BF_10_ = 0.098
	**GFP**	*r* = −0.006 BF_10_ = 0.089	*r* = 0.051 BF_10_ = 0.115	*r* = −0.064 BF_10_ = 0.133	*r* = 0.005 BF_10_ = 0.089	*r* = −0.011 BF_10_ = 0.090	*r* = 0.103 BF_10_ = 0.252	*r* = −0.034 BF_10_ = 0.100	*r* = −0.044 BF_10_ = 0.108	*r* = 0.045 BF_10_ = 0.108	*r* = −0.081 BF_10_ = 0.169
**Microstate G**	**Dur**	*r* = −0.024 BF_10_ = 0.094	*r* = 0.018 BF_10_ = 0.092	*r* = −0.114 BF_10_ = 0.315	*r* = 0.025 BF_10_ = 0.095	*r* = −0.090 BF_10_ = 0.195	*r* = 0.203 BF_10_ = 5.284	*r* = 0.030 BF_10_ = 0.097	*r* = −0.039 BF_10_ = 0.104	*r* < 0.001 BF_10_ = 0.089	*r* = −0.029 BF_10_ = 0.097
	**Occ**	*r* = 0.075 BF_10_ = 0.153	*r* = 0.023 BF_10_ = 0.094	*r* = 0.139 BF_10_ = 0.580	*r* = 0.011 BF_10_ = 0.090	*r* = 0.046 BF_10_ = 0.109	*r* = −0.068 BF_10_ = 0.140	*r* = 0.055 BF_10_ = 0.120	*r* = 0.044 BF_10_ = 0.108	*r* = 0.024 BF_10_ = 0.094	*r* = 0.060 BF_10_ = 0.126
	**Cov**	*r* = 0.083 BF_10_ = 0.174	*r* = 0.042 BF_10_ = 0.106	*r* = 0.096 BF_10_ = 0.217	*r* = 0.041 BF_10_ = 0.105	*r* = −0.023 BF_10_ = 0.094	*r* = 0.047 BF_10_ = 0.110	*r* = 0.068 BF_10_ = 0.140	*r* = 0.042 BF_10_ = 0.105	*r* = 0.021 BF_10_ = 0.093	*r* = 0.074 BF_10_ = 0.151
	**GFP**	*r* = −0.010 BF_10_ = 0.090	*r* = 0.047 BF_10_ = 0.110	*r* = −0.052 BF_10_ = 0.116	*r* = −0.015 BF_10_ = 0.091	*r* = −0.024 BF_10_ = 0.094	*r* = 0.135 BF_10_ = 0.523	*r* = 0.017 BF_10_ = 0.092	*r* = −0.030 BF_10_ = 0.097	*r* = 0.044 BF_10_ = 0.108	*r* = −0.058 BF_10_ = 0.123

Blue squares indicate correlations with substantial-to-strong level of evidence. Strong coefficients at BF_10_ > 10 are underlined.

## Data Availability

The data presented in this study is available on request from the corresponding author. The data are not publicly available due to privacy restrictions.

## Supplementary Materials

The following are available online at https://www.mdpi.com/article/10.3390/jpm11111216/s1, Table S1: The results of two-way ANOVA for the duration parameter; Table S2: The results of two-way ANOVA for the occurrence parameter; Table S3: The results of two-way ANOVA for the duration parameter; Table S4: The results of two-way ANOVA for the GFP parameter; Table S5: Bayesian Pearson Correlation coefficients between age and ARSQ dimensions; Table S6: Bayesian Pearson correlation coefficients between age and microstate parameters. Figure S1: Plot of spatial correlations between microstate C, when *k* = 4 (Top), and microstates C and F, when *k* = 7 (Middle and Bottom).
