# Supplementary material for: Relationship between Spatiotemporal Dynamics of the Brain at Rest and Self-Reported Spontaneous Thoughts: An EEG Microstate Approach
Source: J Pers Med. 2021 Nov 17;11(11):1216. doi: 10.3390/jpm11111216 (PMC8625384; doi:10.3390/jpm11111216)
Supplement: Supplementary file 1 [file jpm-11-01216-s001.zip › jpm-1440058-supplementary.pdf]

Journal of
Personalized Medicine

MDPI

Supplementary Materials

Table S1. The results of two-way ANOVA for the duration parameter.

**Duration**				
	**F(df)**		**p**	**η^2p**
Age	F(1, 194)	3.926	**0.049**	0.020
Gender	F(1, 194)	4.380	**0.038**	0.022
Microstate	F(6, 194)	2.292	0.089	0.012
Microstate × Age	F(6, 194)	1.254	0.289	0.006
Microstate × Gender	F(6, 194)	1.232	0.296	0.006

Significant results are highlighted in bold.

Table S2. The results of two-way ANOVA for the occurrence parameter.

**Occurrence**				
	**F(df)**		**p**	**η^2p**
Age	F(1, 194)	4.432	**0.037**	0.022
Gender	F(1, 194)	3.377	0.068	0.017
Microstate	F(6, 194)	5.908	**<0.0005**	0.030
Microstate × Age	F(6, 194)	0.816	0.523	0.004
Microstate × Gender	F(6, 194)	1.921	0.099	0.010

Significant results are highlighted in bold.

Table S3. The results of two-way ANOVA for the duration parameter.

**Coverage**				
	**F(df)**		**p**	**η^2p**
Age	F(1, 194)	0.591	0.443	0.003
Gender	F(1, 194)	0.953	0.330	0.005
Microstate	F(6, 194)	3.921	**0.006**	0.020
Microstate × Age	F(6, 194)	0.774	0.524	0.004
Microstate × Gender	F(6, 194)	1.815	0.135	0.009

Significant results are highlighted in bold.

Table S4. The results of two-way ANOVA for the GFP parameter.

**GFP**				
	**F(df)**		**p**	**η^2p**
Age	F(1, 194)	0.327	0.568	0.002
Gender	F(1, 194)	9.620	**0.002**	0.047
Microstate	F(6, 194)	6.018	**<0.0005**	0.030
Microstate × Age	F(6, 194)	1.584	0.189	0.008
Microstate × Gender	F(6, 194)	3.291	**0.018**	0.017

Significant results are highlighted in bold.

Table S5. Bayesian Pearson correlation coefficients between age and ARSQ dimensions.

		**DoM**	**ToM**	**Self**	**Planning**	**Sleepi-ness**	**Comfort**	**SA**	**HC**	**Vis**	**VT**
Age	*r*	-0.178	**-0.193**	-0.174	-0.103	-0.147	0.147	0.061	**-0.198**	-0.080	-0.185
	BF_{10}	1.967	**3.520**	1.727	0.251	0.733	0.736	0.128	**4.258**	0.167	2.530

Correlations with substantial evidence are highlighted in bold.

Table S6. Bayesian Pearson correlation coefficients between age and microstate parameters.

		MS A	MS B	MS C	MS D	MS E	MS F	MS G
				**Duration**				
Age	*r*	0.095	0.181	0.132	**0.201**	0.172	0.132	0.173
	BF_{10}	0.215	2.189	0.482	**4.852**	1.620	0.493	1.699
				**Occurrence**				
Age	*r*	**-0.224**	-0.096	-0.159	-0.091	-0.091	-0.115	-0.149
	BF_{10}	**12.761**	0.216	1.065	0.200	0.198	0.319	0.789
				**Coverage**				
Age	*r*	-0.137	0.061	0.038	0.031	0.024	-0.022	-0.031
	BF_{10}	0.550	0.127	0.102	0.098	0.094	0.093	0.098
				**GFP**				
Age	*r*	-0.033	0.006	-0.004	-0.003	-1.836e-5	-0.016	-0.025
	BF_{10}	0.099	0.090	0.089	0.089	0.089	0.092	0.095

Correlations with substantial evidence are highlighted in bold.

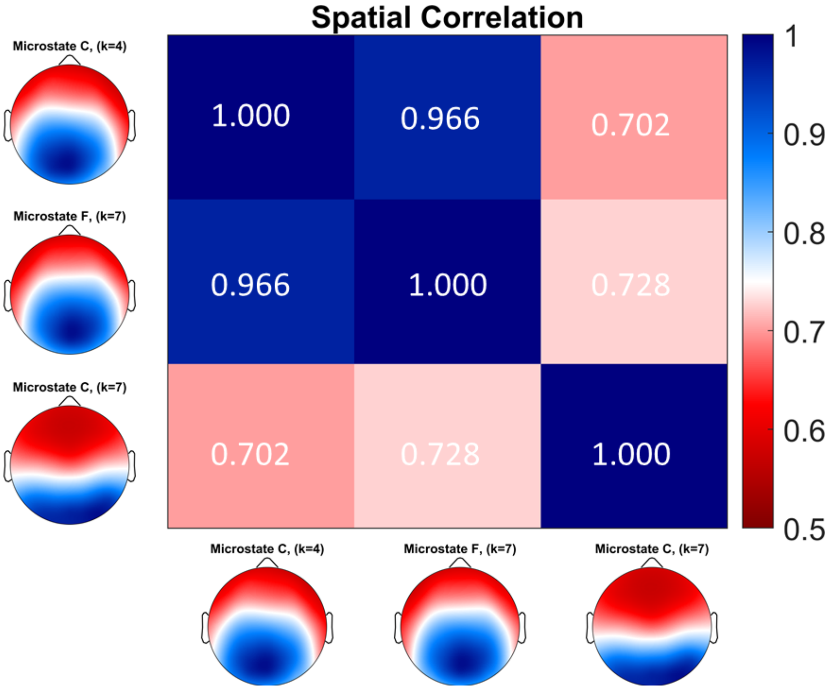

Figure S1. Plot of spatial correlations between microstate C, when $k = 4$ (Top), and microstates C and F, when $k = 7$ (Middle and Bottom). All topographies yielded a high correlation values ($r > 0.7$). Microstate C, when k=4 and microstate F, when $k = 7$, had a very high spatial correlation value confirming hypothesis, that spatially similar microstates might be merged into a single microstate, when suboptimal number of clusters is used. Additionally, both topographies had posterior activity and visually were similar to microstate C, reported in study by Pipinis et al. [16].
